# Severe Preeclampsia and Oliguric Acute Tubular Necrosis Following Placental Abruption in a Dichorionic Diamniotic Twin Pregnancy

**DOI:** 10.7759/cureus.103228

**Published:** 2026-02-08

**Authors:** Rachel Kohler, Sanjana Nalla, Sahith Kaki, Prashant Obed R Dundi

**Affiliations:** 1 Medicine, Lake Erie College of Osteopathic Medicine, Erie, USA; 2 Obstetrics and Gynecology, Lifeline Medical Associates, Edison, USA; 3 Family Medicine, Allegheny Health Network, Pittsburgh, USA

**Keywords:** acute kidney injury, dichorionic diamniotic twins, magnesium levels, placental abruption, preeclampsia

## Abstract

Preeclampsia remains a leading cause of maternal and perinatal mortality, largely attributed to abnormal placentation, incomplete spiral artery remodeling, and increased antiangiogenic factors. Established risk factors for preeclampsia include a history of preeclampsia, chronic hypertension, diabetes, obesity, advanced maternal age, and multiple gestation. This report details a case of a 31-year-old with a history of five total pregnancies, two term pregnancies, one preterm pregnancy, two induced abortions, and one living child (G5P2121) with a dichorionic diamniotic twin pregnancy who presented at 24 weeks and two days of gestation with severe preeclampsia and placental abruption of Twin A. Following emergent repeat low transverse cesarean, the patient developed evidence of reversible disseminated intravascular coagulation (DIC) and subsequent oliguric acute tubular necrosis (ATN). Aggressive blood pressure control with multiple intravenous antihypertensives led to improvement in urine output by postoperative day one and steady recovery of renal function throughout her hospitalization. Our case highlights the importance of early recognition and prompt management of hypertension in high-risk pregnancies, as well as the need for close surveillance in patients with significant risk factors, especially those with multiple gestations. Prompt blood pressure control and multidisciplinary care can avert renal replacement therapy in oliguric ATN complicating preeclampsia.

## Introduction

Preeclampsia is a clinical condition in pregnancy characterized by new-onset hypertension greater than 140 mmHg systolic blood pressure (SBP) and 90 mmHg diastolic blood pressure (DBP) after 20 weeks of gestation, accompanied by proteinuria exceeding 300 mg in 24 hours or a urine protein-to-creatinine ratio of 0.3 or higher. The threshold for severe range pressures is an SBP greater than 160 mmHg and a DBP greater than 110 mmHg. It affects approximately 2% to 8% of pregnancies worldwide and continues to be a major culprit of both maternal and perinatal morbidity and mortality [1]. The pathophysiology is theorized to occur in two stages, with abnormal placentation and incomplete spiral artery remodeling in early gestation being the first, and an increase in antiangiogenic factors in the latter stages of pregnancy. The combination of these stages leads to placental ischemia, ultimately causing systemic vascular dysfunction, which can present in the mother as elevated blood pressures, visual disturbances, and proteinuria, and as a result, potentially contribute to hypoxia and fetal growth restriction in the baby [1,2]. Significant risk factors include a history of preeclampsia, multiple gestation, chronic hypertension, obesity, diabetes, and advanced maternal age [2].

Acute tubular necrosis (ATN) is the most common cause of intrinsic acute kidney injury (AKI), resulting from ischemic damage to the renal tubular epithelial cells. Clinical manifestations include oliguria and a rapidly rising creatinine with an increase of greater than 0.3 within 48 hours, a creatinine increase greater than 1.5 times baseline within seven days, or a decrease in urine output less than 0.5 mL/kg/h for six hours, as defined by the Kidney Disease Improving Global Outcomes (KDIGO) [3]. Common insults for the development of this condition include volume depletion, nephrotoxic agents, radiocontrast agents, and trauma. During pregnancy, susceptibility to ATN is due to many factors, including female gender, advanced age, anemia, hemorrhage, and hypertensive disorders, such as preeclampsia [3]. All of these contributors carry large implications for both maternal and fetal outcomes.

The intertwining of preeclampsia and ATN is clinically significant, as ATN in the setting of pregnancy is relatively uncommon. However, preeclampsia, hemolysis, elevated liver enzymes, low platelet count (HELLP) syndrome, and placental abruption, among other conditions that cause vascular abnormalities, can predispose patients to ischemic renal injury, leading to oliguric ATN or even necrosis, which is important for clinicians to take into consideration, as the consequences can be profound. In one cohort study of women with preeclampsia and oliguric renal failure, the need for dialysis was approximately 10%, with the perinatal mortality reaching close to 40% [4]. Therefore, the importance of prompt detection and management in these situations is crucial for identifying precipitating factors and treating the underlying cause. This case highlights oliguric ATN in the setting of preeclampsia, emphasizing the pathophysiological interplay, diagnostic challenges, and management considerations.

## Case presentation

A 31-year-old patient, with five total pregnancies, two term pregnancies, one preterm pregnancy, two induced abortions, and one living child (G5P2121) at 24 weeks and two days of gestation with a dichorionic diamniotic twin pregnancy, presented to the emergency department with abdominal cramping and vaginal bleeding. Her past medical history was significant for preeclampsia in prior pregnancies, obesity, and sickle cell trait. The patient had inconsistent prenatal care, with limited prenatal visits and no close follow-up with maternal-fetal medicine despite her history of preeclampsia. Four blood pressure readings less than 140/90 mmHg were documented during her prenatal visits. The patient did not receive any antihypertensives in the prenatal period but was appropriately prescribed low-dose aspirin per guidelines. 

On admission, fetal heart tones were absent in Twin A. The patient's blood pressure was elevated at this time, with an initial reading of 139/93 mmHg. The patient subsequently underwent emergent repeat low transverse cesarean delivery with bilateral salpingectomy for suspected placental abruption. The delivery was complicated by the intrauterine fetal demise of Twin A, while Twin B was transferred to the neonatal intensive care unit (NICU). Estimated blood loss was 1000 mL. Intraoperatively, she received 1500 mL of IV fluids and one unit of fresh frozen plasma (FFP). Pathological examination of the placenta confirmed abruption of Twin A.

**Figure 1:**
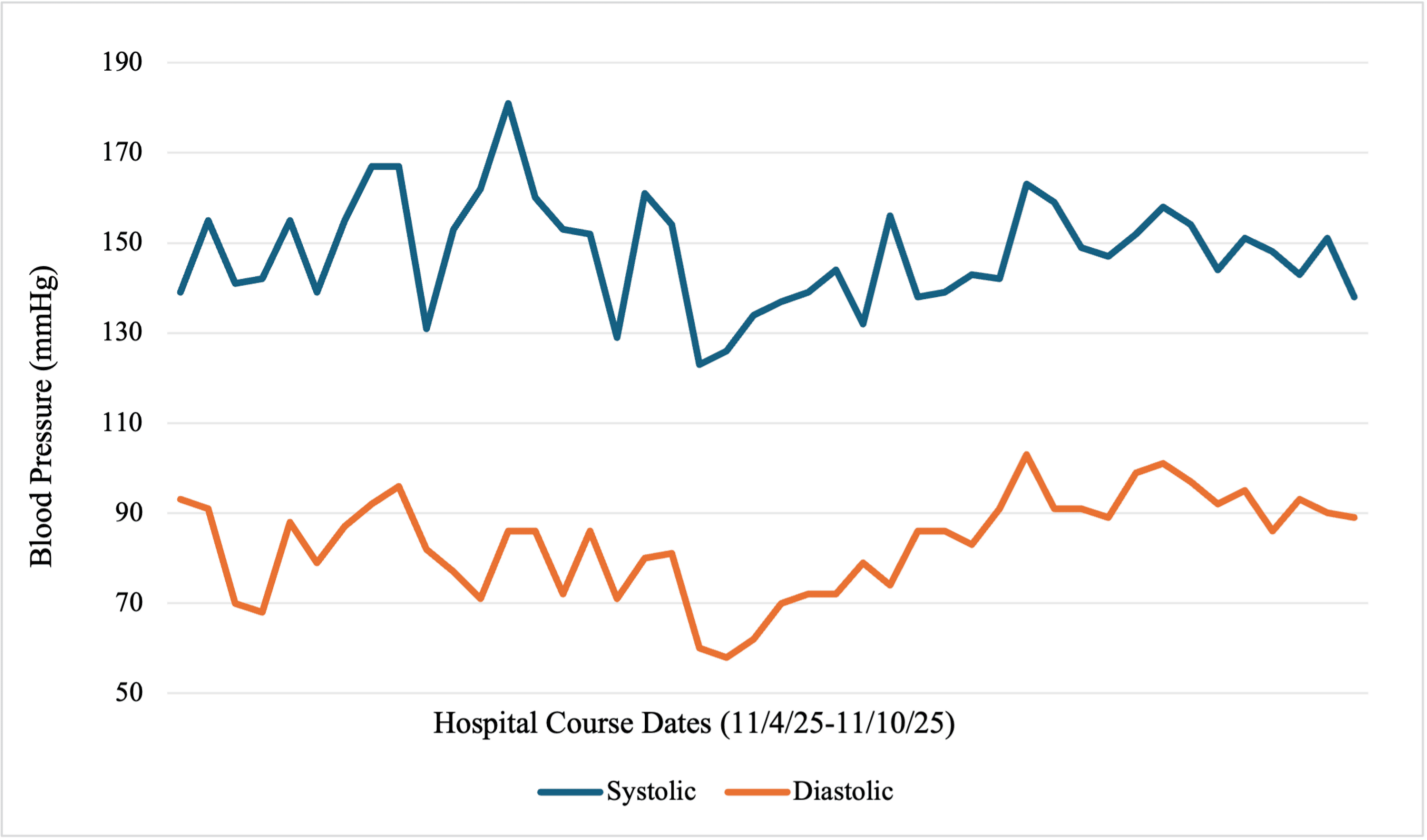
Our patient’s blood pressure trend from hospital admission to discharge Multiple measurements were taken each day, given her history of preeclampsia and current clinical status. The graph emphasizes the patient’s continued elevated pressures, peaking at 181/86 mmHg on her second day of admission, despite intervention with antihypertensives during her hospital course. The pressures gradually decreased on the latter end of her stay.

**Figure 2:**
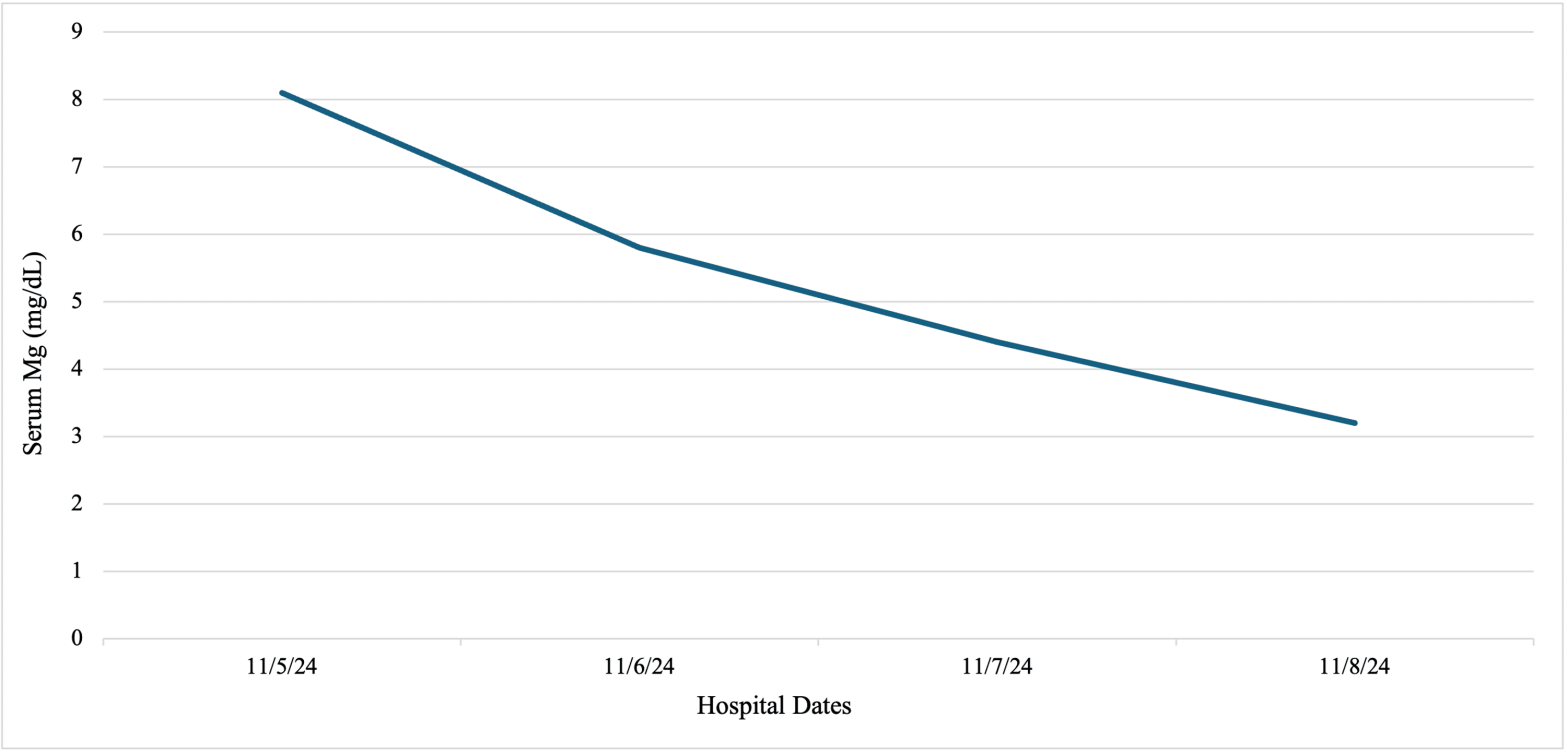
Our patient’s blood pressure trend from hospital admission to discharge Our patient’s serum magnesium (Mg) levels from admission through the days before discharge; the peak level reached 8.1 mg/dL on hospital day 2 before gradually declining to 3.2 mg/dL, which remained above the normal range. Mg toxicity may have contributed to her clinical presentation and is also recognized as a potential risk factor for the development of acute tubular necrosis.

**Figure 3:**
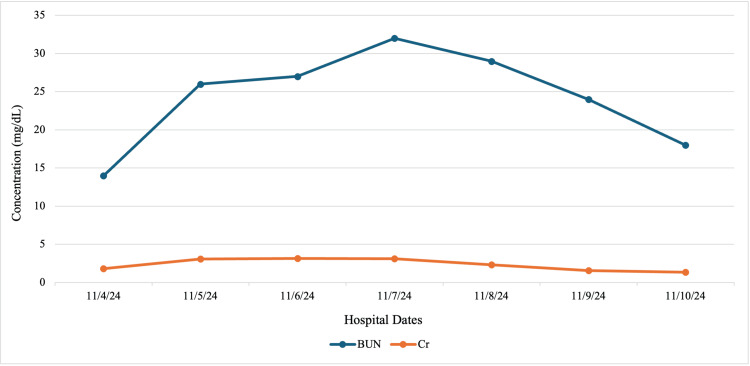
Our patient’s creatinine (Cr) and blood urea nitrogen (BUN) concentrations throughout her stay The peak levels for her creatinine and BUN were 3.17 mg/dL and 32, respectively, on days 2 and 3 of admission.

Postoperatively, the patient developed persistent severe-range blood pressures, reading in the 160s/90s mmHg, despite multiple antihypertensive medications, as shown in Table 1 and Figure 1. She was additionally initiated on magnesium sulfate but developed toxicity requiring discontinuation, peaking at 7.9 mg/dL, as seen in Table 2 and Figure 2. She was transferred to the intensive care unit (ICU) for further blood pressure management with a nicardipine drip. Her course was complicated by AKI with oliguria (0.37 mL/kg/hr), with a rise in creatinine from 0.8 to 2.8 mg/dL, high anion gap metabolic acidosis requiring bicarbonate infusion, and nephrotic-range proteinuria (urine protein/creatinine ratio 4.8 mg/g). With blood pressure stabilization, urine output began to improve on postoperative day (POD) 1, as noted in Table 3 and Figure 3. These collective findings were consistent with ATN in the setting of preeclampsia with severe features.

**Table 1:** Our patient's blood pressure measurements taken throughout each day of her hospital course

Hospital Dates	Systolic Blood Pressure/ Diastolic Blood Pressure (mmHg)	Hospital Dates	Systolic Blood Pressure/ Diastolic Blood Pressure (mmHg)	Reference Systolic Blood Pressure/ Diastolic Blood Pressure (mmHg)
11-04-2024	139/93	11-07-2024	137/70	<120/<80
11-04-2024	155/91	11-07-2024	139/72	
11-04-2024	141/70	11-07-2024	144/72	
11-04-2024	142/68	11-07-2024	132/79	
11-04-2024	155/88	11-07-2024	156/74	
11-05-2024	139/79	11-08-2024	138/86	
11-05-2024	155/87	11-08-2024	139/86	
11-05-2024	167/92	11-08-2024	143/83	
11-05-2024	167/96	11-08-2024	142/91	
11-05-2024	131/82	11-08-2024	163/103	
11-05-2024	153/77	11-08-2024	159/91	
11-05-2024	162/71	11-09-2024	149/91	
11-05-2024	181/86	11-09-2024	147/89	
11-06-2024	160/86	11-09-2024	152/99	
11-06-2024	153/72	11-09-2024	158/101	
11-06-2024	152/86	11-09-2024	154/97	
11-06-2024	129/71	11-09-2024	144/92	
11-06-2024	161/80	11-10-2024	151/95	
11-06-2024	154/81	11-10-2024	148/86	
11-06-2024	123/60	11-10-2024	143/93	
11-06-2024	126/58	11-10-2024	151/90	
11-07-2024	134/62	11-10-2024	138/89	

**Table 2:** Our patient's serum magnesium levels throughout her hospital course

Hospital Dates	Patient’s Serum Magnesium (mg/dL)	Reference Serum Magnesium (mg/dL)
11-05-2024	7.9	1.6-2.6
11-05-2024	8.1	
11-05-2024	7.2	
11-06-2024	5.8	
11-07-2024	4.4	
11-08-2024	3.2	

**Table 3:** Our patient's serum creatinine and blood urea nitrogen levels taken throughout her hospital course

Hospital Dates	Creatinine (mg/dL)	Blood Urea Nitrogen (mg/dL)	Blood Urea Nitrogen Reference Range (mg/dL)	Creatinine reference range (mg/dL)
11-04-2024	0.89	10	6-20	0.5-0.9
11-04-2024	1.83	15		
11-05-2024	2.62	20		
11-05-2024	2.85	22		
11-05-2024	2.91	24		
11-05-2024	3.09	26		
11-06-2024	3.17	27		
11-07-2024	3.13	32		
11-08-2024	2.33	29		
11-09-2024	1.58	24		
11-10-2024	1.35	18		

She also developed anemia (hemoglobin 6.7 g/dL; normal 12-16 g/dL), thrombocytopenia (platelets ~111 × 10³/µL; normal 150-450 × 10³/µL), elevated prothrombin and fibrin products, and decreased fibrinogen (120 mg/dL; normal 200-400 mg/dL), raising high suspicion for placental abruption-induced non-overt disseminated intravascular coagulation (DIC). Lack of clinical signs such as bleeding and thrombosis, along with gradually progressive changes in laboratory findings, pointed away from overt DIC. She was transfused with two units of packed red blood cells during her admission. Leukocytosis (peak white blood cell count of 35 × 10³/µL; normal 4-11 × 10³/µL) prompted empiric ceftriaxone for suspected urinary tract infection, though cultures were negative. Additional complications included hyponatremia (nadir 124 mmol/L; normal 135-145 mmol/L), mild pulmonary edema, and a viral upper respiratory infection (rhinovirus). A subsequent echocardiogram showed preserved ejection fraction and no structural abnormalities.

By PODs 2-3, urine output had significantly improved (greater than 4 L/day), renal ultrasound excluded obstruction, and creatinine began to downtrend. Nicardipine was weaned, and she was transitioned to oral antihypertensives. Laboratory indices gradually stabilized, and acidosis resolved.

By PODs 4-5, renal function was improving (creatinine of 3.1 to 1.6 mg/dL), electrolytes normalized, and the urine protein-to-creatinine ratio decreased from 4.8 g/g to 0.8 g/g. Blood pressure remained elevated but stable on oral agents. Nephrology cleared the patient for discharge from a renal standpoint. 

By POD 6, she was clinically stable: ambulating, tolerating a diet, and voiding spontaneously. Laboratory studies showed creatinine of 1.58 mg/dL, hemoglobin of 9.5 g/dL, and a platelet count of 125k. She was discharged on nifedipine XL 90 mg daily, labetalol 300 mg every eight hours, and iron supplementation, with instructions for close follow-up with obstetrics, nephrology, primary care, and cardiology.

## Discussion

Our patient's presentation highlights a rare but severe cascade involving severe preeclampsia, placental abruption, and subsequent oliguric ATN in a dichorionic diamniotic pregnancy. Compared with singleton pregnancies, twin pregnancies develop preeclampsia earlier, have more severe presentations, and require more antihypertensive agents [5]. Clinical guidelines for the management of hypertensive disorders do not currently differ between single and multiple gestations despite the increased risk associated with twin pregnancies [5]. 

Our patient had a known history of preeclampsia in a prior singleton pregnancy and obesity and was of African American descent, further compounding her risk. While not consistently observed across all studies, sickle cell trait, as was present in our patient, has been shown to increase the risk of preeclampsia in pregnancy, particularly in already high-risk populations, including those with multifetal gestation [6,7]. Despite the multitude of significant risk factors in our patient, she did not receive extensive prenatal care and had not been seen by maternal-fetal medicine before presentation. While the patient was appropriately prescribed low-dose aspirin, she appears to have had limited surveillance of her blood pressure, with only four documented blood pressure readings less than 140/90 mmHg during her prenatal visits in the second trimester. While the optimal frequency of blood pressure monitoring in patients at high risk for preeclampsia is not completely agreed upon, the Working Group recommends blood pressure monitoring every two weeks until 20 weeks of gestation and weekly thereafter in these individuals [8].

Additionally, self-monitoring of blood pressure is recommended to allow for increased frequency of monitoring [8]. Self-monitoring also enables the integration of telemedicine in prenatal care for these patients, providing increased access and convenience of care [8]. Our patients' limited surveillance demonstrates an important area for improvement in early detection. Additionally, telemedicine and self-monitoring of blood pressure were not utilized as adjuncts in her care, showing another possible addition to the standard care of patients at high risk for preeclampsia.

Following placental abruption of Twin A, our patient developed DIC and ATN, further complicating her management. Hemorrhage, including placental abruption, is a well-recognized prerenal cause of AKI, stemming from depletion of intravascular volume and reduced renal perfusion pressure, resulting in ischemic tubular injury. The release of tissue factor during abruption can additionally lead to DIC, where renal blood flow can be further impaired [8]. Likely, both factors contributed to ATN in our patient. Oliguric ATN, as seen in our patient, is associated with a worse prognosis than non-oliguric ATN due to complications including a higher risk of volume overload, hyperkalemia, uremia, and need for renal replacement therapy [9, 10]. Her clinical course demonstrates the possible downstream consequences of abruption in the setting of preeclampsia and the importance of multidisciplinary involvement in the care of these patients, including obstetrics, nephrology, and critical care. Our patient recovered with aggressive blood pressure control, including multiple IV antihypertensives and a nicardipine drip. She did not require renal replacement therapy or develop any additional complications of her ATN, demonstrating the importance of early and aggressive blood pressure management in maternal outcomes.

## Conclusions

Early recognition of preeclampsia in high-risk patients, such as those with multiple gestations, is essential and relies heavily on strong prenatal care with close monitoring during pregnancy. Poor prenatal care in patients such as ours with a multitude of significant risk factors can delay both diagnosis and intervention, consequently increasing the risk of adverse outcomes such as placental abruption, DIC, and ATN. We suggest the use of telemedicine and self-monitoring as important adjuncts to more frequent prenatal visits to improve surveillance and access to care. Our patient presented with placental abruption and developed subsequent DIC and oliguric ATN, a rare but recognized complication of preeclampsia and abruption. Aggressive blood pressure control in this patient, as well as a multidisciplinary approach to her care, resulted in recovery of renal function without the need for renal replacement therapy. Given our patients' relatively rapid improvement, we encourage early addition of multiple antihypertensive measures and continued vigilance in patients with preeclampsia and abruption.
